# Characterization of Heterogeneous Prostate Tumors in Targeted *Pten* Knockout Mice

**DOI:** 10.1371/journal.pone.0147500

**Published:** 2016-01-25

**Authors:** Hanneke Korsten, Angelique C. J. Ziel-van der Made, Wytske M. van Weerden, Theo van der Kwast, Jan Trapman, Petra W. Van Duijn

**Affiliations:** 1 Department of Pathology, Josephine Nefkens Institute, Erasmus Medical Center, Rotterdam, The Netherlands; 2 Department of Urology, Josephine Nefkens Institute, Erasmus Medical Center, Rotterdam, The Netherlands; Florida International University, UNITED STATES

## Abstract

Previously, we generated a preclinical mouse prostate tumor model based on PSA-Cre driven inactivation of *Pten*. In this model homogeneous hyperplastic prostates (4-5m) developed at older age (>10m) into tumors. Here, we describe the molecular and histological characterization of the tumors in order to better understand the processes that are associated with prostate tumorigenesis in this targeted mouse *Pten* knockout model. The morphologies of the tumors that developed were very heterogeneous. Different histopathological growth patterns could be identified, including intraductal carcinoma (IDC), adenocarcinoma and undifferentiated carcinoma, all strongly positive for the epithelial cell marker Cytokeratin (CK), and carcinosarcomas, which were negative for CK. IDC pattern was already detected in prostates of 7–8 month old mice, indicating that it could be a precursor stage. At more than 10 months IDC and carcinosarcoma were most frequently observed. Gene expression profiling discriminated essentially two molecular subtypes, denoted tumor class 1 (TC1) and tumor class 2 (TC2). TC1 tumors were characterized by high expression of epithelial markers like Cytokeratin 8 and E-Cadherin whereas TC2 tumors showed high expression of mesenchyme/stroma markers such as Snail and Fibronectin. These molecular subtypes corresponded with histological growth patterns: where TC1 tumors mainly represented adenocarcinoma / intraductal carcinoma, in TC2 tumors carcinosarcoma was the dominant growth pattern. Further molecular characterization of the prostate tumors revealed an increased expression of genes associated with the inflammatory response. Moreover, functional markers for senescence, proliferation, angiogenesis and apoptosis were higher expressed in tumors compared to hyperplasia. The highest expression of proliferation and angiogenesis markers was detected in TC2 tumors. Our data clearly showed that in the genetically well-defined *PSA-Cre;Pten-loxP/loxP* prostate tumor model, histopathological, molecular and biological heterogeneity occurred during later stages of tumor development.

## Introduction

Prostate tumor development is a multistep process in which prostate cells acquire malignant characteristics by the accumulation of genetic and epigenetic alterations [1, 2]. Many biological processes, including sustained proliferative signaling, induction of angiogenesis and cell death resistance can play a role during tumorigenesis [3]. Complementary, the role of the tumor microenvironment (TME) has emerged as an important determinant in prostate tumor development and progression [4]. Moreover, the inflammatory response might contribute to the regulation of these biological processes by releasing a wide range of cytokines, chemokines, growth factors, survival factors and proangiogenic factors to the TME [5, 6].

Human prostate cancer is a heterogeneous disease, which displays a variety of histopathological tumor growth patterns and molecular abnormalities [7–9]. The intratumoral heterogeneity of prostate cancer significantly challenges the development of effective treatment strategies. Although prostate tumors can be classified by expression profiling into subtypes with a distinct prognosis [10–13], little is known about the mechanisms by these different tumor subtypes develop. Both biological and molecular processes might contribute to tumor heterogeneity. Moreover, differences in clonal evolution and differences in tumor initiating cells are postulated to explain tumor heterogeneity [14–17]. In a clinical setting, study of the dynamics of prostate tumor development is impossible. Therefore, well-defined preclinical model systems are very helpful in unraveling mechanisms of tumor development including tumor heterogeneity.

*PTEN* inactivation is one of the most frequent genetic alterations in prostate cancer [18, 19]. Several genetically engineered mouse prostate tumor models (GEMMs) based on targeted bi-allelic deletion of the *Pten* tumor suppressor gene have been developed, which all resemble to a certain extent the several stages of human prostate cancer [20–27]. However, none of the initial publications on mouse prostate tumor models based on *Pten* inactivation described tumor heterogeneity.

Previously, we described that upon biallelic loss of *Pten* in the PSA-Cre targeted *Pten* knockout model, clearly defined stages of prostate hyperplasia and cancer develop, while minimal pathologic changes were found upon mono-allelic loss of *Pten* [23]. We studied the initial stages of hyperplasia in the PSA-Cre targeted *Pten* knockout model and identified Clu+Tacstd2+Sca1+ luminal epithelial progenitor cells as candidate tumor initiating cells [28]. In the present study we focus on the characterization of tumor heterogeneity and the identification of biological and molecular processes associated with tumor development in the *PSA-Cre;PtenLoxP/LoxP* mouse prostate cancer model. Tumors were found to be histologically heterogeneous, but two main separate histological growth patterns, intraductal carcinoma (IDC) and carcinosarcoma, could be discriminated. Based on gene expression profiling the heterogeneous tumors could be separated in two molecular subtypes, denoted tumor class 1 (TC1) and tumor class 2 (TC2), corresponding to a distinct prostate tumor histology. Main biological processes that were associated with prostate tumor development were an increased inflammatory response, senescence, proliferation, angiogenesis and apoptosis.

## Materials and Methods

### Generation of Prostate Targeted Pten Knockout Mice

PSA-Cre mice (strain FVB), mice carrying the *Pten-loxP* allele (strain 129Ola) and *PSA-Cre;Pten-loxP/loxP* mice have been described previously [23]. All *PSA-Cre;Pten-loxP/loxP* mice had a mixed 129/FVB genetic background. Cre negative littermates were kept as controls. Mice were housed according to institutional guidelines, procedures were carried out in compliance with the standards for use of laboratory animals and all efforts were made to minimize suffering. Animal experiments performed in this manuscript have been approved by the animal experimental committee of the Erasmus Medical Center (DEC-consult, permit number 106-05-11).

### RNA extraction, cDNA preparation and QPCR analysis

From normal and hyperplastic prostates individual lobes (anterior, ventral, dorsal and lateral) were dissected separately. From each mouse, pieces of the four individual lobes were pooled and RNA was extracted from this pool. In mice with large prostate tumors where the individual lobes could not be distinguished pieces of different regions of the tumor were pooled. RNA was isolated from snap frozen mouse prostates using the Qiagen Easy RNA isolation Kit (Qiagen, Hilden, Germany) according to the manufacturer’s guidelines, including an on column DNAseI digestion. RNA quality was assessed using the RNA 6000 Nano kit in a 2100 Bioanalyser (Agilent, Santa Clara, CA).

For reverse transcription 2 μg total RNA was incubated for 1 h at 37°C in buffer containing 50mM Tris-HCl pH 8.3, 75 mM KCl, 3mM MgCl_2_, 10mM DTT and 1mM dNTPs, supplemented with 400 U M-MLV-reverse transcriptase (Thermo Fisher Scientific, Waltham, MA), 80 U RNAguard (Amersham Biosciences, Little Chalfont, UK) and 1 μg oligodT primer. The procedure for QPCR analysis was described earlier [28]. Primer sequences for both PCR as QPCR are provided in S1 Table.

### Probe preparation and hybridization, and analysis of Affymetrix gene expression arrays

Five μg total RNA was used to prepare antisense biotinylated RNA according to the manufacturer’s one-cycle protocol (Affymetrix, Santa Clara, CA). Hybridization to Affymetrix Mouse Genome 430 2.0 GeneChips (>39000 transcripts), staining, washing and the scanning procedures were performed by ErasmusMC Center for Biomics according to the Affymetrix protocol. The Affymetrix gene expression data were normalized based on the average signal intensity. Before transforming expression array data to log2 values, low expression values (<4) were set at 4. For unsupervised hierarchical clustering and visualization of genes with the highest differential expression the programs Cluster and Treeview [29] were used. In addition, by performing Significance Analysis of Microarrays (SAM) [30] gene expression relative differences to the standard deviation of these expression levels within one group was calculated. Microarray data are available in the ArrayExpress database (www.ebi.ac.uk/arrayexpress) under accession number E-MTAB-3970.

### Immunohistochemical analysis

Procedures for immunohistochemistry were described earlier [23, 28]. Detailed antibody information is provided in S2 Table.

### Statistics

GraphPad Prism v5.01 (GraphPad Software, San Diego, CA) was used for statistical analysis. The Mann-Whitney two-tailed *t*-test was used for comparison between groups and a *p* value <0.05 (*) was considered significant.

## Results

### Prostate tumors of targeted *Pten* knockout mice are heterogeneous

**Fig 1:**
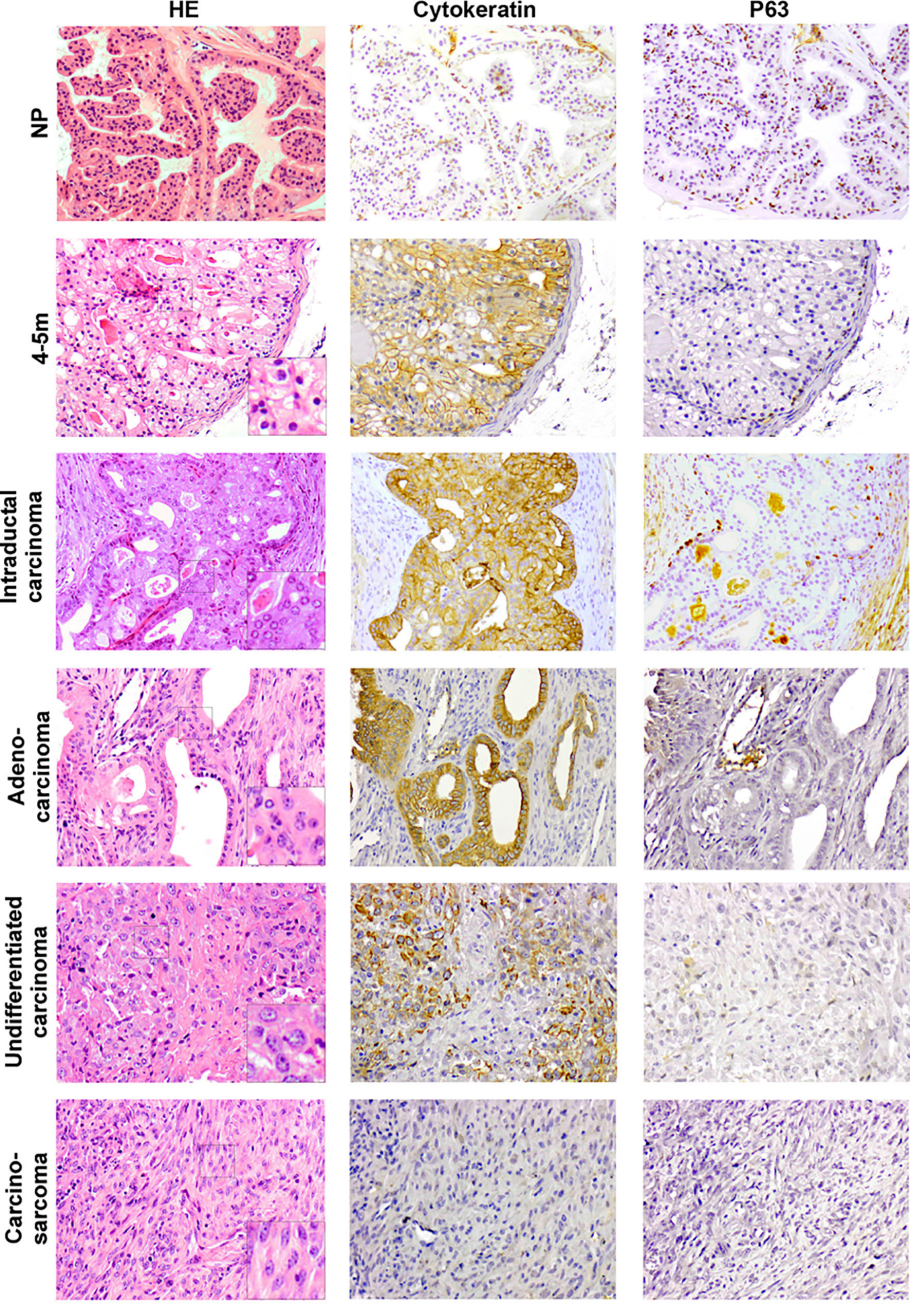
Characterization of hyperplastic prostates and prostate tumors of targeted *Pten* knockout mice. The prostate histology (HE staining) of the anterior lobe of hyperplastic prostates (4-5m) and different growth patterns (IDC, adenocarcinoma, undifferentiated carcinoma and carcinosarcoma) in prostate tumors (> 10m) are shown (Magnification: 200x). Normal prostate (NP) is included as a reference. A more detailed picture of the nuclear structures in these prostates is shown at higher magnifications. To characterize the prostate cells consecutive slides stained for P63 and Cytokeratin are shown for each growth pattern.

Previously, we described prostate tumor development in *PSA-Cre;Pten-loxP/loxP* mice [23]. At 4–5 months the lumen of the prostate glands of targeted bi-allelic *Pten* knockout mice were filled with homogeneous, cytokeratin (CK) positive hyperplastic epithelial cells lined by P63+ basal epithelial cells (Fig 1). Clu+Tacstd2+Sca-1+ luminal epithelial progenitor cells were identified as candidate tumor initiating cells [28]. At older age (>10m) all mice developed invasive prostate tumors. In the present study, the tumors are characterized and processes associated with tumor development are identified.

Although hyperplastic prostates (HP) were histologically very homogeneous, considerable heterogeneity was only detected in the prostate tumors. In contrast to the regular nucleï of hyperplastic cells at 4-5m, tumor cells showed nuclear atypia with prominent nucleoli (Fig 1). Already at 7-8m foci of cells with atypical nucleï could be detected, which were named intraductal carcinoma (IDC), because of the histologic resemblance to human IDC [31] (S1 Fig). Prostates with characteristics of IDC contained dysplastic cells that showed a cribriform growth pattern surrounded by an interrupted P63+ basal epithelial cell layer (Fig 1, S1 Fig).

At >10m all mice displayed heterogeneous prostate tumors containing areas with distinct histopathological growth patterns. Three types of carcinoma could be distinguished: IDC, adenocarcinoma and undifferentiated carcinoma (Fig 1). Tumor cells in IDC, adenocarcinoma and undifferentiated carcinoma showed strong CK staining. Carcinosarcoma, composed of spindle shaped cells, was the fourth growth pattern that could be detected in the heterogeneous prostate tumors. Carcinosarcoma cells were negative for CK staining. IDC and carcinosarcoma were the dominant tumor types. Because foci of IDC were already detected at 7-8m, IDC might represent a precursor stage of the other tumor types. However, transitions from one growth pattern to another were not apparent.

### Molecular subclasses of prostate tumors in PSA-Cre targeted *Pten* knockout mice, identified by gene expression profiling, match prostate tumor histology

**Fig 2:**
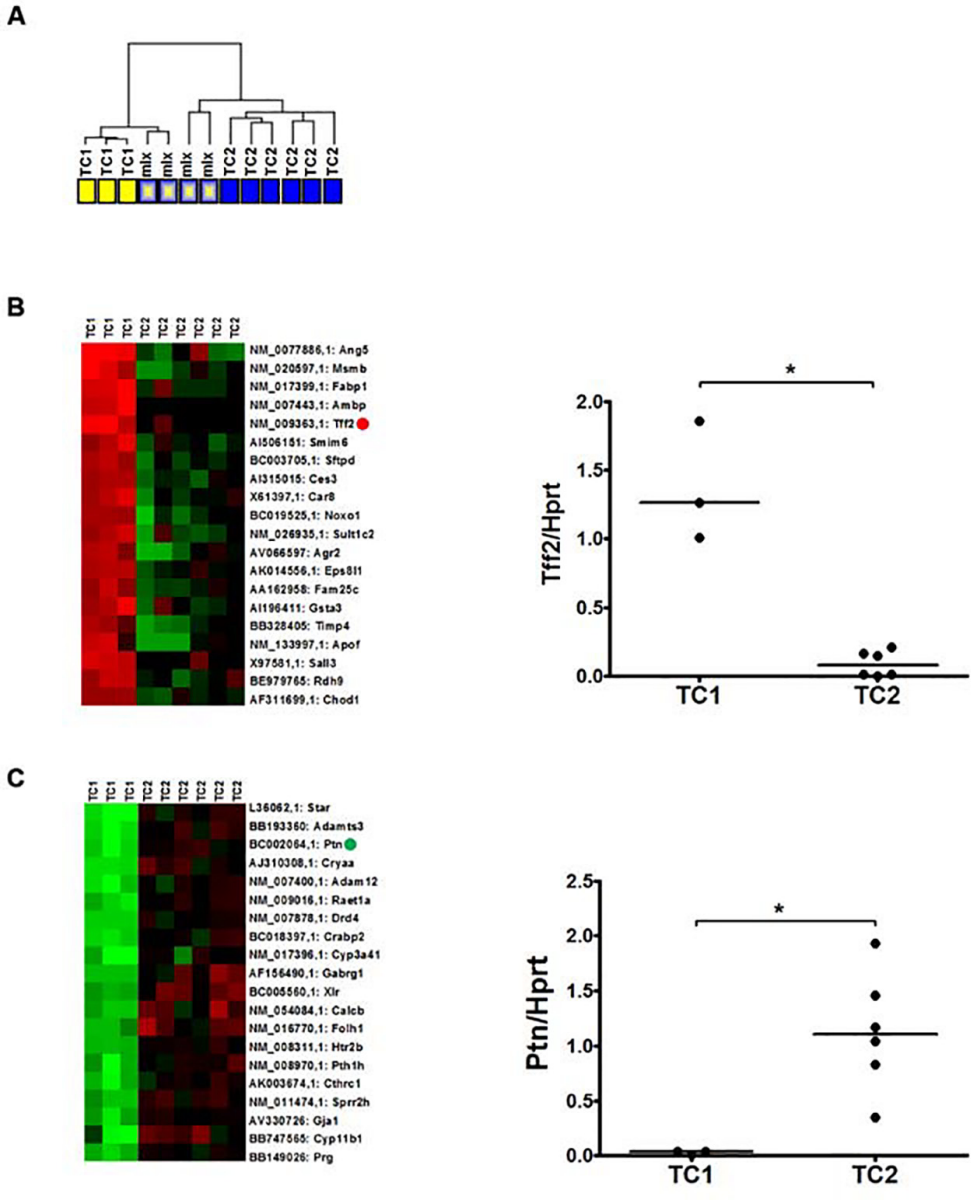
Two molecular subclasses of prostate tumors in PSA-Cre targeted *Pten* knockout mice match major prostate tumor growth patterns (A) Unsupervised hierarchical clustering of global gene expression data of thirteen tumor samples. Yellow and blue boxes indicated TC1 and TC2 tumors respectively. Where TC1 tumors mainly consisted of carcinomas, in TC2 tumors carcinosarcoma was the dominant growth pattern. More heterogeneous tumors are indicated by the dual blue/yellow colored boxes. Top-20 list of genes higher (B) and lower (C) expressed in TC1 (n = 3) tumors as compared to TC2 (n = 6) tumors as identified by SAM. Green indicates lower gene expression and red indicates higher expression. The expression of *Tff2* (indicated by the red dot) and *Ptn* (indicated by the green dot) was confirmed by Q-PCR analysis. Expression *Hprt* was used as Q-PCR reference. * *p* < 0.05, by Mann-Whitney two tailed test.

As described above, prostate tumors were histological heterogeneous. To extend the characterization of the tumors and to gain further insight into the underlying mechanisms of prostate tumorigenesis, global gene expression profiling was undertaken of three normal prostates (NP), three hyperplastic prostate samples (HP) and thirteen prostate tumors. First, unsupervised hierarchical clustering of the thirteen tumor samples was performed, discriminating two main clusters (Fig 2A). To address the question whether the differences in gene expression profiles were associated with prostate tumor histology, these thirteen tumors were independently scored for the presence of different tumor growth patterns by two pathologists. Although all prostate tumor samples showed heterogeneous histopathological growth patterns, global differences could be detected. Of the thirteen tumors, in three tumors the predominating growth pattern (>70%) was carcinoma (IDC, adenocarcinoma and undifferentiated carcinoma). This carcinoma group was denoted as tumor class 1 (TC1) and grouped together in the same cluster (Fig 2A). Six prostate tumors, which grouped together in another cluster (Fig 2A), were dominated by large areas (>70%) of carcinosarcoma and were denoted as tumor class 2 (TC2). Four tumors contained a more complex mixture of carcinoma and carcinosarcoma that hampered the determination of a predominating growth pattern. These four samples were indicated as mixed tumors and were excluded from further analyses (Fig 2A).

Next, SAM was done to identify genes differentially expressed between TC1 and TC2 (Fig 2B and 2C). Among the genes that were highly expressed in TC1 tumors were genes associated with higher expression in epithelial cells, like *Msmb and Tff2*. Genes that are lower expressed in TC1 tumors compared to TC2, such as *Ptn* and *Gja1*, were known to be expressed in stromal/mesenchymal cells [32–35]. The names of the top twenty differentially expressed genes are listed in (S3 Table and S4 Table). As examples, the expression of *Tff2* and *Ptn* in individual prostate tumors, as determined by q-PCR analysis, is shown (Fig 2B and 2C). Thus, although hampered by the heterogeneity of the samples, tumor samples could clearly be separated in TC1 and TC2 subtypes, based on gene expression and histology.

**Fig 3:**
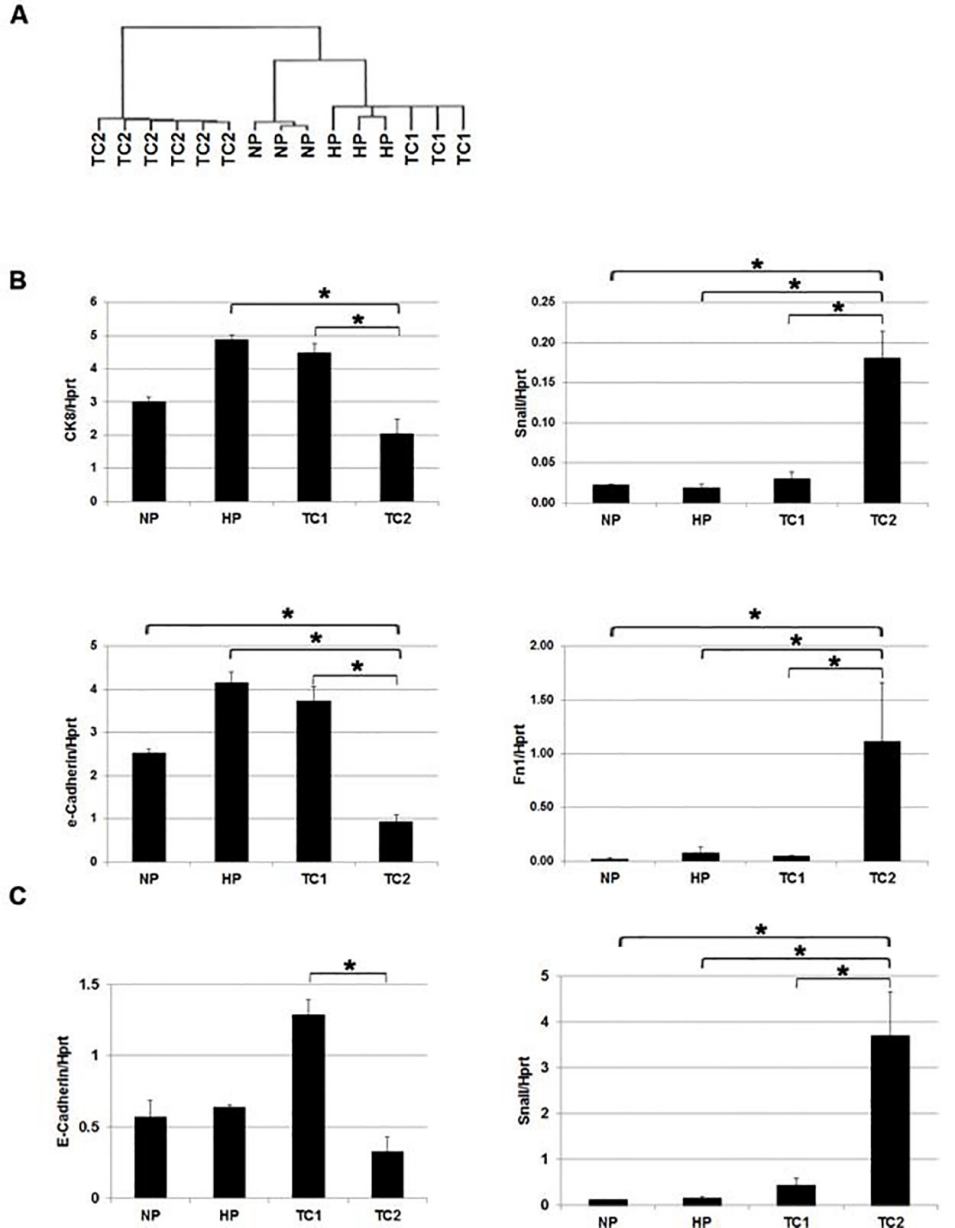
Differential expression of epithelial and mesenchymal markers in TC1 and TC2 tumors. (A) Unsupervised hierarchical clustering of global gene expression data of normal prostates (NP), hyperplastic prostates (HP) and TC1 and TC2 prostate tumors. (B) The relative mRNA expression levels of *CK8*, *E-cadherin*, *Snail* and *Fibronectin* in NP (n = 3), HP (n = 3), TC1 (n = 3) and TC2 (n = 6) as detected in the expression arrays. Indicated gene expressions were plotted relative to the housekeeping gene *Hprt*. (C) Representative Q-PCR analysis of *Snail* and *E-cadherin* expression. Expression of *Hprt* was used as Q-PCR reference. Data are presented as mean +/- SE. * *p* < 0.05, by Mann-Whitney two tailed test.

Next, unsupervised hierarchical clustering of global gene expression data from the tumor samples (TC1, TC2) together with normal prostate (NP) and hyperplasia (HP) was undertaken (Fig 3A). Interestingly, one cluster comprised of only TC2 tumors whereas the more epithelial like TC1 tumors co-clustered with HP samples and to a lesser extend with NP. Further analysis of expression profiles of known markers for epithelial (*Cytokeratin 8* and *E-cadherin*) and mesenchymal (*Snail* and *Fibronectin*) cells confirmed significant differential expression of genes associated with epithelial cells and mesenchymal cells in TC1 and TC2, respectively (Fig 3B). Q-PCR analysis confirmed the observed significantly higher expression of the epithelial marker *E-cadherin* in TC1 tumors and the high expression of the mesenchymal marker *Snail* in TC2 tumors (Fig 3C). Overall, the high mRNA expression of epithelial markers *CK8* and *E-cadherin* in TC1 tumors is in line with the immunohistochemical data for CK expression as observed in Fig 1. Further comparison of expression of individual genes in TC1 and TC2 with HP and NP showed that the relative mRNA expression of the basal epithelial cell marker *P63* was lower in HP and prostate tumors (S2A Fig). The *androgen receptor (AR)* mRNA expression was slightly increased in prostates of targeted *Pten* knockout mice as compared to NP, in contrast to markers for differentiated luminal epithelial cells like *Nkx3*.*1* and *Probasin* (S2A Fig). IHC analysis showed a similar or even slightly higher nuclear AR protein expression in TC1 and TC2 tumors compared to NP and HP (S2B Fig). Compared to NP, the expression of p-Akt, as determined by IHC analysis, was strongly increased in HP and TC1 and TC2 tumors, showing the activation of the Akt signaling pathway by bi-allelic *Pten* inactivation in these samples (S2B Fig).

### Prostate tumors are associated with increased expression of genes associated with an inflammatory response

**Fig 4:**
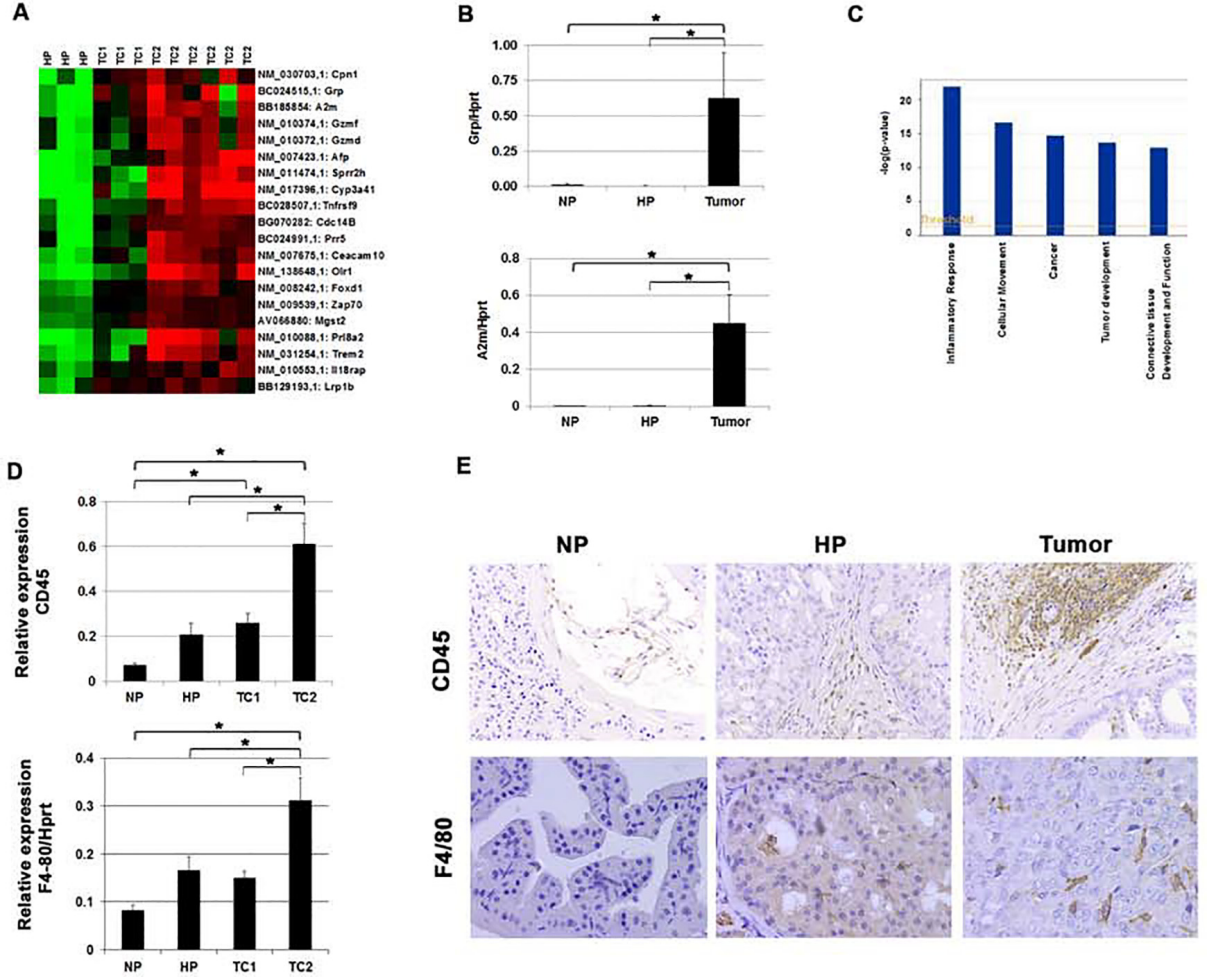
Increased expression of markers associated with inflammatory response in prostate tumors of targeted *Pten* knockout mice. (A) Top-20 list of genes higher expressed in prostate tumors compared to HP as identified by SAM. Green indicates lower expression and red indicates higher expression. (B) Q-PCR analysis of *Grp* and *A2m* expression in NP (n = 3), HP (n = 3) and prostate tumors (n = 9). Expression of *Hprt* was used as Q-PCR reference. Data are given as mean +/- SE. (C) Top-5 processes annotated by Ingenuity analysis based on genes differentially expressed between HP and prostate tumors. (D) Relative RNA expression analysis of *CD45* and *F4/80* in NP (n = 3), HP (n = 3) and TC1 (n = 3) and TC2 (n = 6) prostate tumors. Indicated CD45 and F4/80 expression was plotted relative to the housekeeping gene *Hprt*. Data are presented as mean +/- SE. (E) CD45 and F4/80 staining of immune cells in NP, HP and tumor (Magnification: 125x and 200x respectively). * *p* < 0.05 by Mann-Whitney two tailed test.

To extend the biological and molecular characterization of prostate tumors and to identify processes involved in prostate tumor development we carried out SAM to identify genes differentially expressed in all prostate tumors as compared to HP (Fig 4A, S5 Table and S6 Table). In Fig 4A the 20 genes with the highest expression in prostate tumors as compared to HP are visualized. Remarkably, many of the top 20 genes, such as *Grp*, *A2m*, *Tnfrsf9*, *Gzmf*, *Zap70*, *Il18rap* and *Gzmd*, were identified as genes associated with the inflammatory response [36, 37]. As examples q-PCR analyses of *Grp* and *A2m* are shown, confirming the significantly increased expression of these genes in tumor samples compared to NP and HP (Fig 4B). Strong differential expression of genes involved in the inflammatory response between tumor and HP samples was supported by Ingenuity analysis (Fig 4C). Here, inflammatory response was by far the top process associated with prostate tumors.

Further studies showed that RNA expression of the common leukocyte marker CD45 was significantly higher in HP and tumor samples as compared to normal prostate (NP) (Fig 4D). Moreover, the highest expression of CD45 mRNA was found in TC2 tumors as compared to HP and TC1 (Fig 4D). Similarly, increased mRNA expression of the macrophage marker F4/80 was observed in HP and tumor samples compared to NP (Fig 4D), with highest expression in TC2 tumors (Fig 4D). Immunohistochemical staining of prostates for CD45 and F4/80 confirmed that both HP and prostate tumors were infiltrated by immune cells that stained positive for the common leukocyte marker and macrophage marker respectively (Fig 4E).

### During prostate tumor development in PSA-Cre *Pten* knockout mice differential expression of markers of senescence, proliferation, angiogenesis and apoptosis was observed

**Fig 5:**
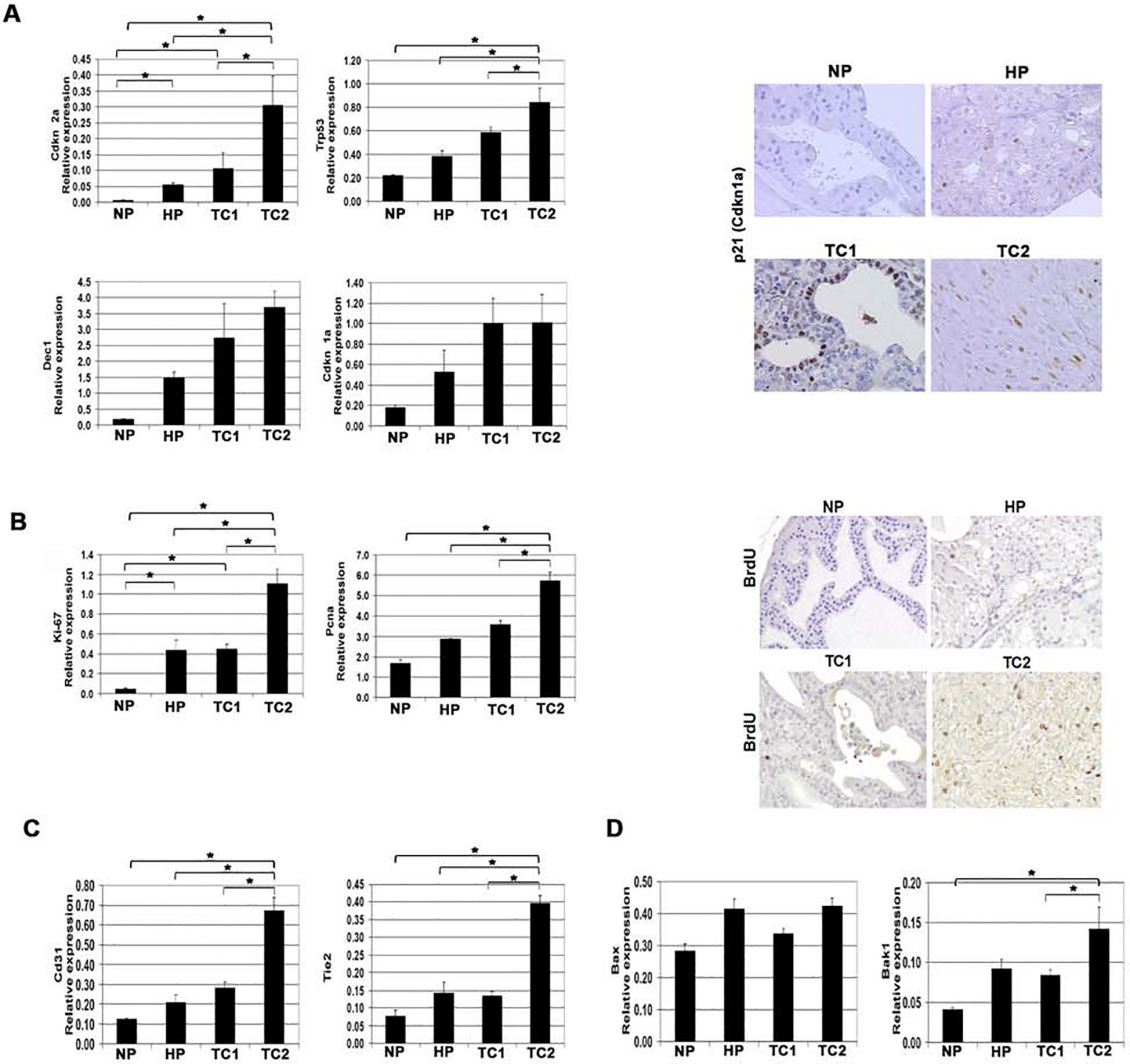
Differential expression of markers associated with senescence, proliferation, angiogenesis and apoptosis in TC1 and TC2 prostate tumors as compared to HP/NP in targeted *Pten* knockout mice. (A) Relative mRNA expression levels of senescence markers *Cdkn1a*, *Trp53*, *Cdkna2a* and *Dec1* in NP, HP, TC1 and TC2 (left panel). Representative p21 immunostaining in tissue slides of the anterior prostate lobe of NP, HP, TC1 and TC2 (right panel). Magnification: 200x. (B) Relative mRNA expression levels of proliferation markers *Ki67* and *Pcna* and representative pictures of BrdU+ cells in anterior prostate lobes of NP, HP, TC1 and TC2 (Magnification: 200x). (C) Relative mRNA expression of angiogenesis markers *CD31* and *Tie2* in NP, HP, TC1 and TC2. (D) Relative mRNA expression of pro-apoptotic markers *Bax* and *Bak1* in NP, HP, TC1 and TC2. The indicated gene expressions were plotted relative to the housekeeping gene *Hprt*. Sample size NP, HP and TC1 (n = 3) and TC2 (n = 6). Data are given as mean +/- SE. * *p* < 0.05 by Mann-Whitney two tailed test.

To further explore which biological processes can be associated with tumor development, RNA expression of markers associated with several processes, like senescence, proliferation and angiogenesis, was analyzed. Previously, Chen et al. reported an increased expression of genes involved in the *Trp53* dependent cellular senescence response in early hyperplasia stages of tumor development in the related Probasin (PB)-Cre targeted *Pten* knockout mouse model [38] We also observed higher expression of *Trp53*, *Trp53*-regulated and *Trp53-*independent senescence markers *Cdkn1a* (encoding p21), *Dec1* and *Cdkn2a* (encoding P16) in HP as compared to NP (Fig 5A). Surprisingly, an even higher mRNA expression level of these markers was observed in prostate tumors, with significantly higher expression of *Cdkn2a* and *Trp53* in TC2 tumors (Fig 5A). The expression of p21 (*Cdkn1a*) was further validated by IHC showing increased expression of p21 protein in HP and tumor samples compared to NP (Fig 5A). Taken together these data indicate that diminished senescence is not a factor involved in tumor development from hyperplasia in the *PSA-Cre;PtenLoxP/LoxP* model.

Two other hallmark biological processes associated with tumor development are proliferation and angiogenesis. In prostates of targeted *Pten* knockout mice an increased proliferation rate was detected, as demonstrated by an elevated expression level of *Ki67* and *Pcna* and the higher number of Bromodeoxyuridine (BrdU) positive cells (Fig 5B). These data extend previous observations [23] that the proliferation rate is increased in HP and tumors as compared to NP. Here we observed that TC2 tumors showed a significantly even higher proliferation rate, as compared to HP and TC1 (Fig 5B). Furthermore, analysis of gene expression showed a higher expression of the endothelium markers *CD31* and *Tie2* in HP and TC1 tumors, and the significantly highest expression of these markers in TC2 tumors (Fig 5C), indicating that angiogenesis is stimulated more in these tumors.

Previously, we published an increased number of cells that stained positive for active Caspase3 in prostate hyperplasia and in tumors in *PSA-Cre;Pten-loxP/loxP* mice [23]. In line with these data, the pro-apoptotic markers *Bax* and *Bak1* were slightly higher expressed in HP and TC1, significantly higher mRNA expression was detected in TC2 tumors, as compared to NP (Fig 5D).

## Discussion

The present study mainly focuses on two aspects of tumor development in the *PSA-Cre;PtenLoxP/LoxP* mouse prostate cancer model [23]: The characterization of tumor heterogeneity, and the identification of biological and molecular processes associated with tumor development. Previously, we showed that the initial stages of hyperplasia development in this model were well-defined and highly homogeneous [28]. The process starts with targeted bi-allelic inactivation of *Pten* in the prostate where the Clu+Tacstd2+Sca1+ luminal epithelial progenitor cells were identified as candidate tumor initiating cells. At 4–5 m a homogeneous prostate hyperplasia filling antecedent prostate glands was seen, but later steps in tumor development are not obvious. Here, we showed that at 7–8 m foci of dysplastic cells have developed. Its morphology strongly resembles that of a neoplastic process seen in a subset of aggressive human prostate cancers, designated IDC. At >10 m all targeted mice developed heterogeneous tumors, with mixed growth patterns characteristic of carcinomas and carcinosarcomas.

Carcinoma in the *PSA-Cre;PtenLoxP/LoxP* mouse prostate cancer model was subdivided in three growth patterns: IDC, adenocarcinoma and undifferentiated carcinoma, with IDC as major component. Additionally, we recognized carcinosarcoma as a dominant growth pattern. This intratumoral heterogeneity complicated detailed molecular characterization of the tumors. This might be a reason why in previous investigations of prostate targeted *Pten* knockout mice, tumors have not been studied in more detail [21, 22, 24–26]. In many of the earlier mouse model studies, pre-malignant prostate intra-epithelial neoplasia (PIN) lesions were defined, which showed morphological resemblance to HP as described in our model [23, 28].

The mouse IDC growth pattern described here is very similar to human IDC and different from human high grade PIN [31]. Accumulating evidence has shown that presence of IDC in human prostate cancers is predictive to the development of high-grade invasive cancer and advanced stage disease [39–44]. Our data, showing foci of IDC at 7-8m, earlier than other growth patterns, indicates the progression of IDC to more rapidly growing epithelial tumors later, but we cannot rule out the option that IDC is an independent growth pattern. Moreover, it can be proposed that IDC and other carcinomas progress to carcinosarcomas by epithelial-mesenchymal transition (EMT). Another aspect that remains to be investigated is the role of (reactive) stroma in tumor development. Because of the heterogeneity of the tumors, identification of reactive stromal cells that were clearly different from carcinosarcoma cells was so far not reliable.

In human prostate cancer, inactivation of one copy of *PTEN* is frequent in early prostate cancer [19]. Complete *PTEN* inactivation can be preferentially demonstrated in late stage aggressive clinical prostate cancer [18]. Importantly, growing data show, like presented here in the *Pten* knockout mouse prostate cancer model, that *PTEN* inactivation in human prostate cancer is frequently associated with IDC [45, 46].

Because of the mixed 129/FVB genetic background in which the *PSA-Cre;PtenLoxP/LoxP* prostate cancer model was generated it might be argued that the observed tumor heterogeneity is due to (small) variation in genetic background between individual mice. Although genetic background can be a determinant in *Pten* knockout mice [27, 47, 48] [Van Duijn et al., manuscript in preparation], intratumoral heterogeneity was detected in every individual mouse. Moreover, we recently generated *PSA-Cre;PtenLoxP/LoxP* mice in a homogeneous FVB genetic background, in which prostate tumors developed showing the same typical heterogeneous histopathological growth patterns (S3 Fig) [van Duijn et al., manuscript in preparation]. It is unlikely that intratumor heterogeneity can be explained by different types of tumor initiating cells as early overexpression of p-Akt, as a marker for Pten inactivation, was exclusively detected in luminal epithelial progenitor cells with identical characteristics [28]. Both secondary genetic alterations and epigenetic processes that can include the microenvironment are candidate processes to contribute to heterogeneity [49]. In fact, recent findings indicate that cooperation between different tumor subtypes can even contribute to tumor growth.

Based on gene expression profiling the heterogeneous prostate tumors could be separated in TC1 and TC2 with differential expression of epithelial and mesenchymal markers, respectively. Due to the complexity, we were unable to identify specific gene expression profiles of the different epithelial growth patterns that are present within TC1. Previously, Wang and co-workers [26] compared expression profiles of four prostate tumors of PB-Cre targeted *Pten* knockout mice with normal prostate, but this number of samples was too small to search for heterogeneity. In their study, among the genes higher expressed in prostate tumors were genes also expressed in the tumor samples described in our study, like *Sppr2h* and *Pgr* (Fig 2 and Fig 3). However, by comparison we observed that many genes reported as higher expressed in prostate tumors of PB-Cre driven *Pten* knockout mice were already overexpressed in HP in the *PSA-Cre;PtenLoxP/LoxP* model [28]. So, it seemed that the tumor samples analyzed previously by Wang et al., [26] were mixtures of HP and TC1, as defined in our study. Other mouse prostate cancer models such as *PB-Fgfr1*, *PB-Myc* and *PSA-Cre;Nkx3*.*1LoxP/LoxP* mice were also subjected to expression profiling, however, also in these studies the number of tumors analyzed was very small, making comparison with the data from the present study unreliable [50–52].

To collect initial information about major biological processes associated with tumor development in the *PSA-Cre;PtenLoxP/LoxP* model, we studied RNA expression of specific markers of inflammation, proliferation, apoptosis, senescence and angiogenesis. Extending data from our previous study [23] we found that cellular proliferation was significantly increased in tumors, but the highest proliferation rate was detected in TC2 samples. Similarly, endothelial cell markers showed significantly higher expression in TC2 samples, indicative of increased angiogenesis. Expression of apoptosis markers was slightly higher in both HP and TC1 tumors and significantly higher in TC2 tumors as compared to NP. Summarizing, these data suggest that TC2 contained the most aggressive tumors, which would be in accordance with the histopathological phenotype.

Remarkably, genes involved in the Trp53 pathway, including senescence markers were the highest expressed in tumors. Previously, it was postulated that senescence regulated by increased Trp53 expression functioned as a barrier for progression from hyperplasia and PIN to tumor in targeted *Pten* knockout mice [38]. Our data show that *Trp53* and senescence markers are even higher expressed in tumors compared to HP indicating a more complex role of these markers in tumor development, but it is evident that downregulation of the Trp53 response is not a prerequisite for tumor development.

Based on gene expression profiles and supported by Ingenuity data and immunohistochemistry, a large difference between NP, HP and tumor samples was found in expression of genes associated with an inflammatory response. It is becoming increasingly clear that inflammatory processes are also associated with human prostate cancer [53–55]. Several studies have reported the association between the presence of specific immune cells and prostate cancer development and progression [55]. For example, tumor-associated macrophages (TAM) might play an important role in tumor initiation and progression. Recent studies reported a higher level of TAM in PIN and prostate cancer compared to benign tissue [56, 57]. Our data in the *PSA-Cre;PtenLoxP/LoxP* mouse prostate cancer model would agree with these findings in human prostate cancer. We did not only observe infiltration of immune cells, including macrophages, in prostate tumors but to a lesser extent also in HP. Immunocompetent GEMMs, like the *PSA-Cre;PtenLoxP/LoxP* model described here, will be excellent tools to further explore the role of inflammation in prostate tumor development.

## Supporting Information

S1 Fig Intraductal carcinoma in prostates of targeted *Pten* knockout mice at 7-8m. An example of the typical histology (HE staining) of IDC in prostates of targeted *Pten* knockout mice at 7-8m is shown. Consecutive sections are stained for P63 and CK. Magnification: 200x.
(EPS)

S2 Fig Analysis of selected genes expressed in of TC1 and TC2 prostate tumors. (A) Relative expression of *P63*, *AR*, *Nkx3*.*1* and *Probasin* mRNA in NP (n = 3), HP (n = 3), TC1 (n = 3) and TC2 (n = 6) tumors. The gene expression was plotted relative to expression of the housekeeping gene *Hprt*. Data are given as mean +/- SE. (B) IHC analysis of AR and p-Akt protein in NP, HP,TC1 and TC2 tumors. Representative pictures of the anterior prostate lobe are shown. Magnification 200x.
(EPS)

S3 Fig Characterization of prostate tumors of targeted Pten knockout mice in the FVB genetic background. The histology (HE staining) of different growth patterns (IDC, adenocarcinoma and carcinosarcoma) in prostate tumors (> 10 m) derived from *PSA-Cre;PtenLoxP/LoxP* mice in the FVB genetic background is shown. Magnification 200 x.
(EPS)

S1 Table Primer sequences used for PCR and QPCR analysis. (DOC)

S2 Table Information of antibodies used for immunohistochemistry. (DOC)

S3 Table Full names of top 20 genes overexpressed in TC1 tumors of *PSA-Cre;Pten-loxP/loxP* mice. (DOC)

S4 Table Full names of top 20 genes overexpressed in TC2 tumors of *PSA-Cre;Pten-loxP/loxP* mice. (DOC)

S5 Table Significantly differentially expressed genes in HP and prostate tumors of *PSA-Cre;Pten-loxP/loxP* mice as assayed by SAM analysis. (DOC)

S6 Table Full names of top 20 genes overexpressed in prostate tumors of *PSA-Cre;Pten-loxP/loxP* mice as assayed by SAM analysis. (DOC)

## References

[1] Jeronimo C, Bastian PJ, Bjartell A, Carbone GM, Catto JW, Clark SJ, et al Epigenetics in prostate cancer: biologic and clinical relevance. Eur Urol. 2011; 60(4): 753–766. 10.1016/j.eururo.2011.06.035 21719191

[2] Taylor BS, Schultz N, Hieronymus H, Gopalan A, Xiao Y, Carver BS, et al Integrative genomic profiling of human prostate cancer. Cancer Cell. 2010; 18(1): 11–22. 10.1016/j.ccr.2010.05.026 20579941 PMC3198787

[3] Hanahan D, Weinberg RA. Hallmarks of cancer: the next generation. Cell. 2011; 144(5): 646–674. 10.1016/j.cell.2011.02.013 21376230

[4] McAllister SS, Weinberg RA. The tumour-induced systemic environment as a critical regulator of cancer progression and metastasis. Nat Cell Biol. 2014; 16(8): 717–727. 10.1038/ncb3015 25082194 PMC6220424

[5] DeNardo DG, Andreu P, Coussens LM. Interactions between lymphocytes and myeloid cells regulate pro- versus anti-tumor immunity. Cancer Metastasis Rev. 2010; 29(2): 309–316. 10.1007/s10555-010-9223-6 20405169 PMC2865635

[6] Grivennikov SI, Greten FR, Karin M. Immunity, inflammation, and cancer. Cell. 2010; 140(6): 883–899. 10.1016/j.cell.2010.01.025 20303878 PMC2866629

[7] Boyd LK, Mao X, Lu YJ. The complexity of prostate cancer: genomic alterations and heterogeneity. Nat Rev Urol. 2012; 9(11): 652–664. 10.1038/nrurol.2012.185 23132303

[8] Fraser M, Berlin A, Bristow RG, van der Kwast T. Genomic, pathological, and clinical heterogeneity as drivers of personalized medicine in prostate cancer. Urol Oncol. 2014; . 24768356 10.1016/j.urolonc.2013.10.020

[9] Squire JA, Park PC, Yoshimoto M, Alami J, Williams JL, Evans A, et al Prostate cancer as a model system for genetic diversity in tumors. Adv Cancer Res. 2011; 112: 183–216. 10.1016/B978-0-12-387688-1.00007-7 21925305

[10] Lapointe J, Li C, Higgins JP, van de Rijn M, Bair E, Montgomery K, et al Gene expression profiling identifies clinically relevant subtypes of prostate cancer. Proc Natl Acad Sci U S A. 2004; 101(3): 811–816. . 14711987 10.1073/pnas.0304146101 PMC321763

[11] Markert EK, Mizuno H, Vazquez A, Levine AJ. Molecular classification of prostate cancer using curated expression signatures. Proc Natl Acad Sci U S A. 2011; 108(52): 21276–21281. 10.1073/pnas.1117029108 22123976 PMC3248553

[12] Schoenborn JR, Nelson P, Fang M. Genomic profiling defines subtypes of prostate cancer with the potential for therapeutic stratification. Clin Cancer Res. 2013; 19(15): 4058–4066. 10.1158/1078-0432.CCR-12-3606 23704282 PMC3732571

[13] Tomlins SA, Mehra R, Rhodes DR, Cao X, Wang L, Dhanasekaran SM, et al Integrative molecular concept modeling of prostate cancer progression. Nat Genet. 2007; 39(1): 41–51. . 17173048 10.1038/ng1935

[14] Boyd LK, Mao X, Xue L, Lin D, Chaplin T, Kudahetti SC, et al High-resolution genome-wide copy-number analysis suggests a monoclonal origin of multifocal prostate cancer. Genes Chromosomes Cancer. 2012; 51(6): 579–589. 10.1002/gcc.21944 22334418

[15] Kobayashi M, Ishida H, Shindo T, Niwa S, Kino M, Kawamura K, et al Molecular analysis of multifocal prostate cancer by comparative genomic hybridization. Prostate. 2008; 68(16): 1715–1724. 10.1002/pros.20832 18781578

[16] Lindberg J, Klevebring D, Liu W, Neiman M, Xu J, Wiklund P, et al Exome sequencing of prostate cancer supports the hypothesis of independent tumour origins. Eur Urol. 2013; 63(2): 347–353. 10.1016/j.eururo.2012.03.050 22502944

[17] Lindberg J, Kristiansen A, Wiklund P, Gronberg H, Egevad L. Tracking the origin of metastatic prostate cancer. Eur Urol. 2015; 67(5): 819–822. 10.1016/j.eururo.2014.09.006 25246081

[18] Robinson D, Van Allen EM, Wu YM, Schultz N, Lonigro RJ, Mosquera JM, et al Integrative clinical genomics of advanced prostate cancer. Cell. 2015; 161(5): 1215–1228. 10.1016/j.cell.2015.05.001 26000489 PMC4484602

[19] Yoshimoto M, Cutz JC, Nuin PA, Joshua AM, Bayani J, Evans AJ, et al Interphase FISH analysis of PTEN in histologic sections shows genomic deletions in 68% of primary prostate cancer and 23% of high-grade prostatic intra-epithelial neoplasias. Cancer Genet Cytogenet. 2006; 169(2): 128–137. . 16938570 10.1016/j.cancergencyto.2006.04.003

[20] De Velasco MA, Tanaka M, Yamamoto Y, Hatanaka Y, Koike H, Nishio K, et al Androgen deprivation induces phenotypic plasticity and promotes resistance to molecular targeted therapy in a PTEN-deficient mouse model of prostate cancer. Carcinogenesis. 2014; 35(9): 2142–2153. 10.1093/carcin/bgu143 24986896 PMC4146423

[21] Kwak MK, Johnson DT, Zhu C, Lee SH, Ye DW, Luong R, et al Conditional deletion of the Pten gene in the mouse prostate induces prostatic intraepithelial neoplasms at early ages but a slow progression to prostate tumors. PLoS One. 2013; 8(1): e53476 10.1371/journal.pone.0053476 23308230 PMC3540073

[22] Luchman HA, Benediktsson H, Villemaire ML, Peterson AC, Jirik FR. The pace of prostatic intraepithelial neoplasia development is determined by the timing of Pten tumor suppressor gene excision. PLoS One. 2008; 3(12): e3940 10.1371/journal.pone.0003940 19081794 PMC2597775

[23] Ma X, Ziel-van der Made AC, Autar B, van der Korput HA, Vermeij M, van Duijn P, et al Targeted biallelic inactivation of Pten in the mouse prostate leads to prostate cancer accompanied by increased epithelial cell proliferation but not by reduced apoptosis. Cancer Res. 2005; 65(13): 5730–5739. . 15994948 10.1158/0008-5472.CAN-04-4519

[24] Ratnacaram CK, Teletin M, Jiang M, Meng X, Chambon P, Metzger D. Temporally controlled ablation of PTEN in adult mouse prostate epithelium generates a model of invasive prostatic adenocarcinoma. Proc Natl Acad Sci U S A. 2008; 105(7): 2521–2526. 10.1073/pnas.0712021105 18268330 PMC2268169

[25] Trotman LC, Niki M, Dotan ZA, Koutcher JA, Di Cristofano A, Xiao A, et al Pten dose dictates cancer progression in the prostate. PLoS Biol. 2003; 1(3): E59 . 14691534 10.1371/journal.pbio.0000059 PMC270016

[26] Wang S, Gao J, Lei Q, Rozengurt N, Pritchard C, Jiao J, et al Prostate-specific deletion of the murine Pten tumor suppressor gene leads to metastatic prostate cancer. Cancer Cell. 2003; 4(3): 209–221. . 14522255 10.1016/s1535-6108(03)00215-0

[27] Svensson RU, Haverkamp JM, Thedens DR, Cohen MB, Ratliff TL, Henry MD. Slow disease progression in a C57BL/6 pten-deficient mouse model of prostate cancer. Am J Pathol. 2011; 179(1): 502–512. 10.1016/j.ajpath.2011.03.014 21703427 PMC3123867

[28] Korsten H, Ziel-van der Made A, Ma X, van der Kwast T, Trapman J. Accumulating progenitor cells in the luminal epithelial cell layer are candidate tumor initiating cells in a Pten knockout mouse prostate cancer model. PLoS One. 2009; 4(5): e5662 10.1371/journal.pone.0005662 19461893 PMC2680948

[29] Eisen MB, Spellman PT, Brown PO, Botstein D. Cluster analysis and display of genome-wide expression patterns. Proc Natl Acad Sci U S A. 1998; 95(25): 14863–14868. . 9843981 10.1073/pnas.95.25.14863 PMC24541

[30] Tusher VG, Tibshirani R, Chu G. Significance analysis of microarrays applied to the ionizing radiation response. Proc Natl Acad Sci U S A. 2001; 98(9): 5116–5121. . 11309499 10.1073/pnas.091062498 PMC33173

[31] Tsuzuki T. Intraductal carcinoma of the prostate: a comprehensive and updated review. Int J Urol. 2015; 22(2): 140–145. 10.1111/iju.12657 25358604

[32] Jackerott M, Lee YC, Mollgard K, Kofod H, Jensen J, Rohleder S, et al Trefoil factors are expressed in human and rat endocrine pancreas: differential regulation by growth hormone. Endocrinology. 2006; 147(12): 5752–5759. . 16973727 10.1210/en.2006-0601

[33] Orr B, Vanpoucke G, Grace OC, Smith L, Anderson RA, Riddick AC, et al Expression of pleiotrophin in the prostate is androgen regulated and it functions as an autocrine regulator of mesenchyme and cancer associated fibroblasts and as a paracrine regulator of epithelia. Prostate. 2011; 71(3): 305–317. 10.1002/pros.21244 20812209 PMC3045659

[34] Solan JL, Hingorani SR, Lampe PD. Changes in connexin43 expression and localization during pancreatic cancer progression. J Membr Biol. 2012; 245(5–6): 255–262. 10.1007/s00232-012-9446-2 22729649 PMC3518378

[35] Thielen JL, Volzing KG, Collier LS, Green LE, Largaespada DA, Marker PC. Markers of prostate region-specific epithelial identity define anatomical locations in the mouse prostate that are molecularly similar to human prostate cancers. Differentiation. 2007; 75(1): 49–61. . 17244021 10.1111/j.1432-0436.2006.00115.x

[36] Chu CT, Pizzo SV. alpha 2-Macroglobulin, complement, and biologic defense: antigens, growth factors, microbial proteases, and receptor ligation. Lab Invest. 1994; 71(6): 792–812. . 7528831

[37] Petronilho F, Danielski LG, Roesler R, Schwartsmann G, Dal-Pizzol F. Gastrin-releasing peptide as a molecular target for inflammatory diseases: an update. Inflamm Allergy Drug Targets. 2013; 12(3): 172–177. . 23621446 10.2174/1871528111312030003

[38] Chen Z, Trotman LC, Shaffer D, Lin HK, Dotan ZA, Niki M, et al Crucial role of p53-dependent cellular senescence in suppression of Pten-deficient tumorigenesis. Nature. 2005; 436(7051): 725–730. . 16079851 10.1038/nature03918 PMC1939938

[39] Chen Z, Chen N, Shen P, Gong J, Li X, Zhao T, et al The presence and clinical implication of intraductal carcinoma of prostate in metastatic castration resistant prostate cancer. Prostate. 2015; 75(12): 1247–1254. 10.1002/pros.23005 25917338

[40] Henry PC, Evans AJ. Intraductal carcinoma of the prostate: a distinct histopathological entity with important prognostic implications. J Clin Pathol. 2009; 62(7): 579–583. 10.1136/jcp.2009.065003 19246509

[41] O'Brien C, True LD, Higano CS, Rademacher BL, Garzotto M, Beer TM. Histologic changes associated with neoadjuvant chemotherapy are predictive of nodal metastases in patients with high-risk prostate cancer. Am J Clin Pathol. 2010; 133(4): 654–661. 10.1309/AJCP8EL5FTZSOBIH 20231619 PMC3047497

[42] Robinson BD, Epstein JI. Intraductal carcinoma of the prostate without invasive carcinoma on needle biopsy: emphasis on radical prostatectomy findings. J Urol. 2010; 184(4): 1328–1333. 10.1016/j.juro.2010.06.017 20723921

[43] Siadat F, Sykes J, Zlotta AR, Aldaoud N, Egawa S, Pushkar D, et al Not all gleason pattern 4 prostate cancers are created equal: A study of latent prostatic carcinomas in a cystoprostatectomy and autopsy series. Prostate. 2015; 75(12): 1277–1284. 10.1002/pros.23009 25963383

[44] Wilcox G, Soh S, Chakraborty S, Scardino PT, Wheeler TM. Patterns of high-grade prostatic intraepithelial neoplasia associated with clinically aggressive prostate cancer. Hum Pathol. 1998; 29(10): 1119–1123. . 9781651 10.1016/s0046-8177(98)90423-3

[45] Lotan TL, Gumuskaya B, Rahimi H, Hicks JL, Iwata T, Robinson BD, et al Cytoplasmic PTEN protein loss distinguishes intraductal carcinoma of the prostate from high-grade prostatic intraepithelial neoplasia. Mod Pathol. 2013; 26(4): 587–603. 10.1038/modpathol.2012.201 23222491 PMC3610824

[46] Morais CL, Han JS, Gordetsky J, Nagar MS, Anderson AE, Lee S, et al Utility of PTEN and ERG immunostaining for distinguishing high-grade PIN from intraductal carcinoma of the prostate on needle biopsy. Am J Surg Pathol. 2015; 39(2): 169–178. 10.1097/PAS.0000000000000348 25517949 PMC4293206

[47] Bianchi-Frias D, Pritchard C, Mecham BH, Coleman IM, Nelson PS. Genetic background influences murine prostate gene expression: implications for cancer phenotypes. Genome Biol. 2007; 8(6): R117 . 17577413 10.1186/gb-2007-8-6-r117 PMC2394769

[48] Freeman D, Lesche R, Kertesz N, Wang S, Li G, Gao J, et al Genetic background controls tumor development in PTEN-deficient mice. Cancer Res. 2006; 66(13): 6492–6496. . 16818619 10.1158/0008-5472.CAN-05-4143

[49] McGranahan N, Swanton C. Biological and therapeutic impact of intratumor heterogeneity in cancer evolution. Cancer Cell. 2015; 27(1): 15–26. 10.1016/j.ccell.2014.12.001 25584892

[50] Acevedo VD, Gangula RD, Freeman KW, Li R, Zhang Y, Wang F, et al Inducible FGFR-1 activation leads to irreversible prostate adenocarcinoma and an epithelial-to-mesenchymal transition. Cancer Cell. 2007; 12(6): 559–571. . 18068632 10.1016/j.ccr.2007.11.004

[51] Ellwood-Yen K, Graeber TG, Wongvipat J, Iruela-Arispe ML, Zhang J, Matusik R, et al Myc-driven murine prostate cancer shares molecular features with human prostate tumors. Cancer Cell. 2003; 4(3): 223–238. . 14522256 10.1016/s1535-6108(03)00197-1

[52] Song H, Zhang B, Watson MA, Humphrey PA, Lim H, Milbrandt J. Loss of Nkx3.1 leads to the activation of discrete downstream target genes during prostate tumorigenesis. Oncogene. 2009; 28(37): 3307–3319. 10.1038/onc.2009.181 19597465 PMC2746257

[53] Haverkamp J, Charbonneau B, Ratliff TL. Prostate inflammation and its potential impact on prostate cancer: a current review. J Cell Biochem. 2008; 103(5): 1344–1353. . 17955503 10.1002/jcb.21536

[54] Sfanos KS, De Marzo AM. Prostate cancer and inflammation: the evidence. Histopathology. 2012; 60(1): 199–215. 10.1111/j.1365-2559.2011.04033.x 22212087 PMC4029103

[55] Strasner A, Karin M. Immune Infiltration and Prostate Cancer. Front Oncol. 2015; 5: 128 10.3389/fonc.2015.00128 26217583 PMC4495337

[56] Fujii T, Shimada K, Asai O, Tanaka N, Fujimoto K, Hirao K, et al Immunohistochemical analysis of inflammatory cells in benign and precancerous lesions and carcinoma of the prostate. Pathobiology. 2013; 80(3): 119–126. 10.1159/000342396 23328608

[57] Gollapudi K, Galet C, Grogan T, Zhang H, Said JW, Huang J, et al Association between tumor-associated macrophage infiltration, high grade prostate cancer, and biochemical recurrence after radical prostatectomy. Am J Cancer Res. 2013; 3(5): 523–529. . 24224130 PMC3816972

