# Supplementary material for: Characterization of Heterogeneous Prostate Tumors in Targeted Pten Knockout Mice
Source: PLoS One. 2016 Jan 25;11(1):e0147500. doi: 10.1371/journal.pone.0147500 (PMC4726760; doi:10.1371/journal.pone.0147500)
Supplement: S1 Table — (DOC) [file pone.0147500.s004.doc]

Table S1. Primer sequences used for PCR and QPCR analysis.

| **Gene name** | **Forward primer** | **Reverse primer** | **Application** |
| --- | --- | --- | --- |
| **Pten (exon 5)** | 5’-TTCCTGAAAGTTAGGCTTCT-3’ | 5’-GGGAGAAAACCTGTTTCCCA-3’ | PCR |
| **Myosin** | 5’-TTACGTCCATCGTGGACAGC-3’ | 5’-TGGGCTGGGTGTTAGTCTTA-3’ | PCR |
| **Hprt** | 5’-TCCCTGGTTAAGCAGTACAG-3’ | 5’-TTCCAGTTTCACTAATGACAC-3’ | QPCR |
| **e-Cadherin** | 5’-GGAGGTGGAGAAGAAGACCA-3’ | 5’-TCGCTGTCGGCTGCCTTCA-3’ | QPCR |
| **Snai1** | 5’-CACACGCTGCCTTGTGTCT-3’ | 5’-TATCTCTTCACATCCGAGTG-3’ | QPCR |
| **Tff2** | 5’-GTGTCATGGAAGTGTCAGCT-3’ | 5’-ACGGGTGGCTCGGCAGT-3’ | QPCR |
| **Ptn** | 5’-CCTCAATACCGCCTTGAAGA-3’ | 5’-TTTCCTGGTCCACAGACGGT-3’ | QPCR |
| **Grp** | 5’-CGCCTCTCAGTCTCCAGC-3’ | 5’-TCCCTTCAGCTGGCAGTTC-3’ | QPCR |
| **A2m** | 5’-GGTGCAGCAAGATATTCCAGT-3’ | 5’-CTAAGAACAGGGCTCTGGTG-3’ | QPCR |
