# Supplementary material for: Characterization of Heterogeneous Prostate Tumors in Targeted Pten Knockout Mice
Source: PLoS One. 2016 Jan 25;11(1):e0147500. doi: 10.1371/journal.pone.0147500 (PMC4726760; doi:10.1371/journal.pone.0147500)
Supplement: S2 Table — (DOC) [file pone.0147500.s005.doc]

Table S2. Information of antibodies used for immunohistochemistry.

| Antibody | **Company** | **Product number** | **Dilution** |
| --- | --- | --- | --- |
| BrdU | Biogenex | MU247-UC, clone IIB5 | 1:150 |
| CD45 | DAKO, Glostrup, Denmark | M0701 | 1:200 |
| CK | DAKO, Glostrup, Denmark | M7019 | 1:400 |
| P63 | DAKO, Glostrup, Denmark | M7247 | 1:100 |
| **F4/80** | AbD Serotec, Oxford, UK | MCA497GA, clone A3-1 | 1:100 |
| **P21** | Santa Cruz, Dallas, USA | Sc-6246 | 1:100 |
| **CD8b** | eBioscience, SD, USA | 14-0083, clone H35-17.2 | 1:200 |
| **p-Akt** | CST, MA, USA | #4060 | 1:100 |
| **AR** | Merck Milipore, Darmstadt,  Germany | 06-680 (PG-21) | 1:200 |
| **Goat Anti-Mouse-biotin** | DAKO, Glostrup, Denmark | E0433 | 1:400 |
| **Swine Anti-Rabbit-biotin** | DAKO, Glostrup, Denmark | E0431 | 1:400 |
