# Supplementary material for: Characterization of Heterogeneous Prostate Tumors in Targeted Pten Knockout Mice
Source: PLoS One. 2016 Jan 25;11(1):e0147500. doi: 10.1371/journal.pone.0147500 (PMC4726760; doi:10.1371/journal.pone.0147500)
Supplement: S3 Table — (DOC) [file pone.0147500.s006.doc]

**Table S3. Full names of top 20 genes overexpressed in TC1 tumors of *PSA-Cre;Pten-loxP/loxP* mice.**

| Abbreviation | Gene Name |
| --- | --- |
| **Ang5** | Angiogenin member 5 |
| Msmb | Beta-microseminoprotein |
| **Fabp1** | Fatty acid binding protein 1, liver |
| **Ambp** | Alpha 1 microglobulinbikunin |
| **Tff2** | Trefoil factor 2 |
| **Smim6** | Small integral membrane protein 6 |
| **Sftpd** | Surfactant associated protein D |
| Ces3 | Carboxylesterase 3 |
| **Car8** | Carbonic anhydrase 8 |
| **Noxo1** | NADPH oxidase organizer 1 |
| **Sult1c2** | Sulfotransferase family, cytosolic, 1C, member2 |
| Agr2 | Anterior gradient 2 |
| **Eps8l1** | EPS8-like 1 |
| **Fam25C** | Family with sequence similarity 25, member C |
| Gsta3 | Glutathione S-transferase, alpha 3 |
| **Timp4** | Tissue inhibitor of metalloproteinase 4 |
| **Apof** | Apolipoprotein F |
| Sall3 | Sall-3 like |
| **Rdh9** | Retinol dehydrogenase 9 |
| **Chod1** | Chondrolectin |
