# Supplementary material for: Characterization of Heterogeneous Prostate Tumors in Targeted Pten Knockout Mice
Source: PLoS One. 2016 Jan 25;11(1):e0147500. doi: 10.1371/journal.pone.0147500 (PMC4726760; doi:10.1371/journal.pone.0147500)
Supplement: S4 Table — (DOC) [file pone.0147500.s007.doc]

**Table S4. Full names of top 20 genes overexpressed in TC2 tumors of *PSA-Cre;Pten-loxP/loxP* mice.**

| Abbreviation | Gene Name |
| --- | --- |
| **Star** | Steroidogenic acute regulatory protein |
| Adamts3 | A disintegrin-like and metallopeptidase (reprolysin type) with thrombospondin type 1 motif, 3 |
| **Ptn** | Pleiotrophin |
| **Cryaa** | Alpha-A-crystallin |
| **Adam12** | A disintegrin and metalloprotease domain 12 |
| **Raet1a** | Retinoic acid early transcript 1, alpha |
| **Drd4** | Dopamine receptor 4 |
| Carpb2 | Cellular retinoic acid-bindingprotein 2 |
| **Cyp3a41** | Cytochrome P450, steroid inducible 3a41 |
| **Gabrg1** | Gamma-aminobutyric acid A receptor gamma 1subunit |
| **Xlr** | X-linked lymphocyte-regulated complex |
| Calcb | Calcitonin-related polypeptide, beta |
| **Folh1** | Folate hydrolase |
| **Htr2b** | 5-hydroxytryptamine (serotonin) receptor 2B |
| Pth1h | Parathyroid hormone-like peptide |
| **Cthrc1** | Collagen triple helix repeat containing protein |
| **Sprr2h** | Small proline-rich protein 2H |
| Gja1 | Gap junction membrane channel protein alpha 1 |
| **Cyp11b1** | Cytochrome P450, family 11, subfamily b, polypeptide 1 |
| **Prg** | Progesteron receptor |
