# Supplementary material for: Characterization of Heterogeneous Prostate Tumors in Targeted Pten Knockout Mice
Source: PLoS One. 2016 Jan 25;11(1):e0147500. doi: 10.1371/journal.pone.0147500 (PMC4726760; doi:10.1371/journal.pone.0147500)
Supplement: S5 Table — (DOC) [file pone.0147500.s008.doc]

Table S5. Significantly differentially expressed genes in HP and prostate tumors of *PSA-Cre;Pten-loxP/loxP* mice as assayed by SAM analysis.

Genes higher expressed in prostate tumors as compared to HP (q-value(%) <7)

| **Accession Code** | **Gene Name** | **Score(d)** | **Fold Change** | **q-value(%)** |
| --- | --- | --- | --- | --- |
| NM_017396,1 | Cyp3a41 | 4.447108451 | 695.04619 | 0 |
| BG96196 | UNKNOWN | 4.023867623 | 19.2868022 | 0 |
| BB350820 | UNKNOWN | 3.914327885 | 15.8842405 | 0 |
| BG070282 | Cdc14B | 3.590402662 | 14.9654763 | 0 |
| NM_007675 | Ceacam10 | 3.509129746 | 24.5517087 | 0 |
| NM_138648 | Olr1 | 3.495240026 | 87.657804 | 0 |
| BB185854 | A2m | 3.492528038 | 40.6270629 | 0 |
| NM_007675,1 | Ceacam10 | 3.412031369 | 25.5987903 | 3.782632 |
| NM_030703,1 | Cpn1 | 3.301796273 | 18.0111988 | 3.782632 |
| BB745211 | Gm10451 | 3.178074122 | 25.8455542 | 3.782632 |
| NM_008242,1 | Foxd1 | 3.172342216 | 8.72977056 | 3.782632 |
| BC024515,1 | Grp | 3.1341144 | 59.325864 | 3.782632 |
| NM_007423,1 | Afp | 3.10673406 | 34.569803 | 3.782632 |
| BC028507,1 | Tnfrsf9 | 3.011659252 | 20.458914 | 3.782632 |
| NM_010374,1 | Gzmf | 2.955652279 | 22.47305 | 3.782632 |
| NM_019911 | Tdo2 | 2.938292338 | 64.3889737 | 6.416964 |
| NM_011474,1 | Sprr2h | 2.922613888 | 45.8125846 | 6.416964 |
| BC024991,1 | Prr5 | 2.911517428 | 18.6966944 | 6.416964 |
| BB129193 | Lrp1b | 2.907925763 | 7.59088302 | 6.416964 |
| BB787829 | Defb41 | 2.898868134 | 16.4595738 | 6.416964 |
| NM_009539,1 | Zap70 | 2.896221667 | 5.02988267 | 6.416964 |
| AV066880 | Mgst2 | 2.876928021 | 5.47444121 | 6.416964 |
| NM_010553,1 | Il18rap | 2.875665884 | 7.32648171 | 6.416964 |
| NM_010375,1 | Gzmg | 2.87008917 | 26.6601226 | 6.416964 |
| NM_010088,1 | Prl8a2 | 2.866259102 | 94.978354 | 6.416964 |
| NM_010372,1 | Gzmd | 2.853342688 | 29.0615324 | 6.416964 |
| NM_031254,1 | Trem2 | 2.844917936 | 21.8716249 | 6.416964 |
| AK016255,1 | Speer7-ps1 | 2.844733404 | 17.9142573 | 6.416964 |
| BB233467 | Pik3cd | 2.816908774 | 5.97195213 | 6.416964 |
| NM_010373,1 | Gzme | 2.81201435 | 33.1412293 | 6.416964 |
| NM_019779,1 | Cyp11a | 2.80141199 | 36.0757658 | 6.416964 |
| NM_010373,1 | Gzme | 2.796870754 | 26.5781513 | 6.416964 |
| L25890,1 | Ephb2 | 2.778899073 | 8.54545775 | 6.416964 |
| AI551889 | UNKNOWN | 2.777583556 | 28.7418368 | 6.416964 |
| AJ310308,1 | Cryaa | 2.755483137 | 52.1469898 | 6.416964 |
| BC008152,1 | Casp1 | 2.753533849 | 9.77033301 | 6.416964 |
| NM_031198,1 | Tcfec | 2.733274253 | 13.839046 | 6.416964 |
| BE947483 | Nhsl2 | 2.72038098 | 9.01485744 | 6.416964 |
| BB149026 | Pgr | 2.719421451 | 18.1854659 | 6.416964 |
| NM_010372,1 | Gzmd | 2.712591938 | 21.0176402 | 6.416964 |
| L36062,1 | Star | 2.702566559 | 69.2837018 | 6.416964 |
| NM_009529,1 | Xmr | 2.702376231 | 88.7987542 | 6.416964 |
| AW538452 | Nav3 | 2.70050021 | 6.51163865 | 6.416964 |
| AI849139 | Lemd1 | 2.693111197 | 8.77416089 | 6.416964 |
| BB359887 | Nim1k | 2.678776728 | 17.4057153 | 6.416964 |
| AV205680 | UNKNOWN | 2.663658713 | 11.1354448 | 6.416964 |
| NM_029509,1 | Gbp8 | 2.660232492 | 4.93686635 | 6.416964 |
| BM250782 | Tnfrsf9 | 2.651329233 | 10.3287714 | 6.416964 |

Genes higher expressed in HP as compared to prostate tumors (q-value(%) <7)

| **Accession Code** | **Gene Name** | **Score(d)** | **Fold Change** | **q-value(%)** |
| --- | --- | --- | --- | --- |
| NM_008318,1 | Ibsp | -3.388208524 | 0.02149951 | 5.133571 |
| NM_008932,1 | Prlr-rs1 | -3.376695635 | 0.06625841 | 5.133571 |
| BC024677,1 | Scgb2b27 | -3.335999223 | 0.01242106 | 5.133571 |
| BB701723 | Abpb | -3.24783554 | 0.03826994 | 5.133571 |
| C79967 | Tktl1 | -3.158494822 | 0.05739379 | 5.133571 |
| AI836671 | Mob3b | -3.145306991 | 0.04434046 | 5.133571 |
| BC019528,1 | Best2 | -2.941631121 | 0.04094875 | 6.844762 |
| AF441863,1 | Uts2r | -2.896129324 | 0.09006397 | 6.844762 |
| M22959,1 | Prlr | -2.853141084 | 0.06543926 | 6.844762 |
