# Supplementary material for: Characterization of Heterogeneous Prostate Tumors in Targeted Pten Knockout Mice
Source: PLoS One. 2016 Jan 25;11(1):e0147500. doi: 10.1371/journal.pone.0147500 (PMC4726760; doi:10.1371/journal.pone.0147500)
Supplement: S6 Table — (DOC) [file pone.0147500.s009.doc]

**Table S6. Full names of top 20 genes overexpressed in prostate tumors of *PSA-Cre;Pten-loxP/loxP* mice as assayed by SAM analysis.**

| Abbreviation | Gene Name |
| --- | --- |
| **Cyp3a41** | Cytochrome P450, family 3, subfamily a, polypeptide 41A |
| Cdc14B | Cell division cycle 14 homolog B |
| **Ceacam10** | Carcinoembryonic antigen-related cell adhesion molecule 10 |
| **Olr1** | Oxidized low density lipoprotein (lectin-like) receptor 1 |
| **A2m** | Alpha-2-macroglobulin |
| **Cpn1** | Carboxypeptidase N, polypeptide 1 |
| **Foxd1** | Forkhead box D1 |
| Grp | Gastin-releasing peptide |
| **Afp** | Alpha fetoprotein |
| **Tnfrsf9** | Tumor necrosis factor receptor superfamily, member 9 |
| **Gzmf** | Granzyme F |
| Sprr2h | Small proline-rich protein 2H |
| **Prr5** | Proline rich 5 |
| **Lrp1b** | Low density lipoprotein receptor-related protein 1B |
| Zap70 | Zeta-chain (TCR) associated protein kinase 70kDa |
| **Mgst2** | Microsomal glutathione S-transferase 2 |
| **Il18rap** | Interleukin 18 receptor accessory protein |
| Prl8a2 | Prolactin family 8, subfamily a, member 2 |
| **Gzmd** | Granzyme D |
| **Trem2** | Triggering receptor expressed on myeloid cells 2 |
